# Subfoveal Choroidal Thickness in Central Serous Chorioretinopathy: A Meta-Analysis

**DOI:** 10.1371/journal.pone.0169152

**Published:** 2017-01-11

**Authors:** Guohai Chen, Radouil Tzekov, Wensheng Li, Fangzheng Jiang, Sihong Mao, Yuhua Tong

**Affiliations:** 1 Department of Ophthalmology, Quzhou People’s Hospital, Quzhou, Zhejiang, PR China; 2 Department of Ophthalmology, University of South Florida, Tampa, Florida, United States of America; 3 The Roskamp Institute, Sarasota, Florida, United States of America; 4 Xiamen Eye Center of Xiamen University, Xiamen, Fujian, PR China; Suzhou University, CHINA

## Abstract

**Purpose:**

To evaluate the relationship between subfoveal choroidal thickness (SFCT) and eyes with central serous chorioretinopathy (CSC) versus fellow or control eyes.

**Methods:**

We performed a meta-analysis using databases including PubMed, Embase and ISI Web of Science to find relevant studies. Weighted mean difference (WMD) was calculated for the SFCT in CSC eyes, the unaffected fellow eyes and normal controls.

**Results:**

Twelve studies were selected for this meta-analysis, including 1108 eyes (397 CSC eyes, 228 unaffected fellow eyes and 483 eyes of normal controls). The meta-analysis clearly demonstrated that the subfoveal choiroid of eyes with a clinical presentation of CSC was thickened compared to unaffected fellow eyes (WMD = 52.81, 95% confidence interval (CI), 39.13–66.49, P<0.00001) and was thickened compared to control eyes (WMD = 145.03, 95%CI, 121.33–168.73, P<0.00001). The mean SFCT measurement of the unaffected fellow eyes showed also significantly increased choroidal thickness compared to that of normal control eyes (WMD = 77.20, 95% CI, 44.98–109.42, P<0.00001). Similar results were obtained in a sub-analysis based on the same instrument.

**Conclusion:**

It is demonstrated that SFCT is significantly increased in eyes with clinical manifestation of CSC, and in the clinically non-manifested fellow eyes. These results support the hypothesis that CSC is a bilateral disorder with an initial unilateral clinical presentation.

## Introduction

Central serous chorioretinopathy (CSC) is a retinal disorder characterized by serous retinal detachment and/or retinal pigment epithelial (RPE) detachment, changes most often confined to the macula, and associated with leakage of fluid through the RPE into the subretinal space [1–2]. Although targeted epidemiological studies have not been conducted, it is believed that CSC is a relatively common non-surgical retinopathy and typically affects young to middle-aged adults [3]. The clinical manifestation is typically unilateral at first visit (~96%) [4], with bilateral involvement likely increasing to 20%-40% over time [4–6]. Several studies indicated that men are much more affected than women, with male-to-female ratios reported of up to 8:1 [7].

Two major hypotheses of the pathogenesis of CSC have been proposed: the choroidal dysfunction and the RPE dysfunction [8]. Although CSC was initially thought to originate in the RPE and be caused mainly by its dysfunction, in 1967 Gass proposed that hyperpermeability of the choriocapillaris might be the main cause [9]. In support of this hypothesis, choroidal abnormalities, e.g. diffuse or multifocal choroidal vascular hyperpermeability, have been demonstrated in patients with CSC by indocyanine green angiography (ICGA) [10,11]. This hyperpermeability of the choroidal vessels appears much larger in area compared to the active leaks attributed to the RPE seen in fluorescein angiography, indicating a more widespread choroidal vascular dysfunction. Some studies indicate that genetic background may predispose to CSC and that thick choroids could be one of its phenotypic indicators [2].

While ICGA and some other methods of visualizing the choroid like laser Doppler flowmetry or ultrasound are generally useful for evaluating the vascular anatomy and the functional status or any abnormalities of the choroidal blood vessels, it is difficult to apply a quantitative analysis to its results, something which would be very desirable for early diagnosis, follow-up of progression and estimates of therapeutic efficacy. From that point of view, optical coherence tomography (OCT), and especially its relatively recent modification–spectral-domain OCT (SD-OCT), may be much better suited for quantifying retinal and choroidal changes in CSC. Thus, in 2008 Spaide et al. described a method to obtain images of the choroid using conventional SD-OCT instruments and to evaluate choroidal thickness measurements, which they called “enhanced depth imaging” OCT technique (EDI-OCT). They demonstrated the capabilities of this to visualize the full choroidal depth and measure the choroid thickness in a 5 by 15-degree rectangle centered on the fovea [12]. Since then, using that method many studies reported that the choroid in the subfoveal region in patients with CSC was thicker compared to the subfoveal choroid in eyes of normal control subjects [13–15]. Despite the abundance of literature on this toipic, to the best of our knowledge no meta-analysis have been published focusing on the relationship between subfoveal choroidal thickness (SFCT) in affected eyes of patients with CSC versus SFCT in contralateral (typically unaffected) eyes or eyes of normal control subjects. As recent relevant data appears to be available, we decided to conduct an independent assessment of the literature and to undertake a meta-analysis in order to get a more complete and precise understanding about the status of SFCT in patients with CSC.

## Materials and Methods

### Search Strategy

We conducted searches of PubMed, Embase and ISI Web of Science, using the terms *“choroidal thickness”* and *“central serous chorioretinopathy”*. A manual search was performed by checking the reference lists of original reports and review articles to identify studies not yet included in the computerized databases. The final search was carried out on April 15, 2016. The language was restricted to English.

### Inclusion and Exclusion Criteria

Articles were considered eligible for inclusion in the meta-analysis if the studies met the following inclusion criteria: (1) evaluating the SFCT in patients with CSC, (2) Independent retrospective or prospective association study, and (3) With sufficient available data to estimate WMD with 95% CI. Abstracts from conferences, full texts without raw data available for retrieval, duplicate publications, letters, and review articles were excluded.

### Data Extraction

The data were extracted independently by two reviewers (G.C. and W.L.). Disagreement was resolved by discussion. The information extracted from each study included the authors of each study, the year of reported, information on study design, location of the trial, instruments, number of subjects and SFCT.

### Qualitative Assessment

We assessed quality of included studies by a modified checklist based on the Newcastle-Ottawa Scale (NOS) [16], in which a study was judged on three categories: selection (four items, one star each), comparability (one item, up to two stars), and exposure/outcome (three items, one star each). A nine-point scale of the NOS (range, 0–9 points) has been developed for the evaluation. Studies were defined as high quality if they had more than seven points; as medium quality if they had between four and six points; and as poor quality if they had fewer than four points. Studies with NOS score above 4 points were included in the final analysis.

### Statistical Analysis

The quantitative data were entered into Cochrane Review Manager (RevMan, software version 5.1, Copenhagen, Denmark: The Nordic Cochrane Center, The Cochrane Collaboration, 2011). The WMD was determined for SFCT in the CSC eyes, the unaffected fellow eyes and the eyes of normal controls, outcome was reported with a 95% CI. P<0.05 was considered statistically significant on the test for overall effect. The I^2^ statistic was calculated to assess heterogeneity between studies (P<0.05 was considered representative of significant statistical heterogeneity) [17]. If there was heterogeneity between studies, a random-effects model was applied to the data. Alternatively, a fixed-effects model was used for pooling the data. Begg’s rank correlation test and Egger’s linear regression test were employed to quantitatively assess publication bias (P<0.05 was considered representative of significant statistical publication bias) [18,19].

## Results

### Overall Characteristics of Selected Studies and Quality Assessment

**Fig 1:**
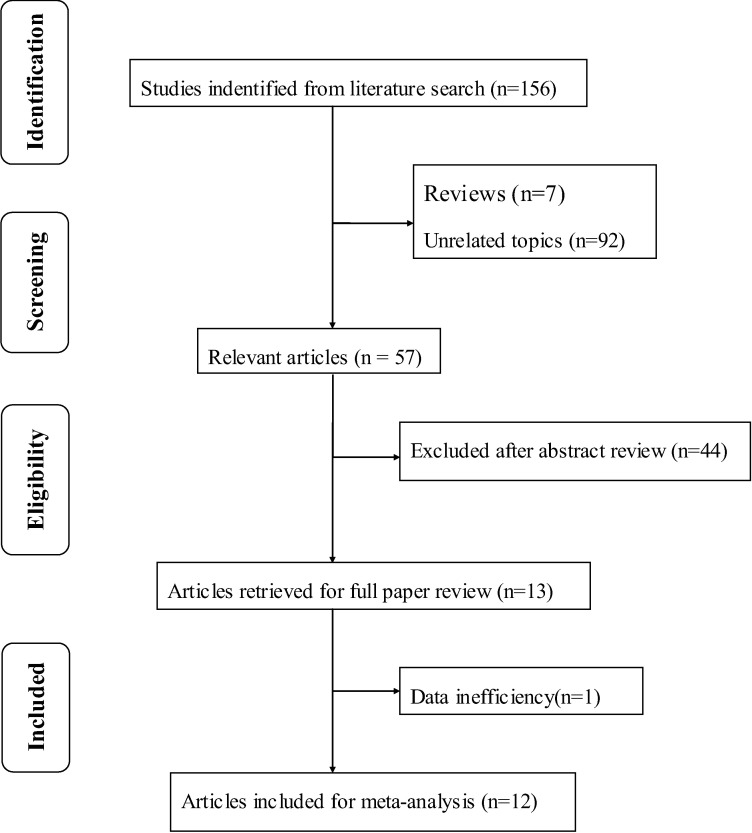
Flow diagram describing selection of studies about association between choroidal thickness and CSC status.

A total of 156 articles were initially identified. Of these, 143 were rejected according to the exclusion criteria and one without sufficient available data[13]. Hence, 12 studies were included in this meta-analysis [14,15,20–29]. Fig 1 provides a flow diagram of the search procedure and results. In total, the results from SFCT measurements in 1108 eyes were analyzed, including measurements from 397 eyes with clinical manifestation of CSC, 228 unaffected fellow eyes and 483 eyes of normal control subjects. According to the NOS used for quality assessment, two studies had moderate quality scores of 6, while ten studies had high quality scores of 7 or 8. The average score of all studies included in the analysis was 6.92. Ten studies employed “standard penetration” SD-OCT instruments using the EDI technique to obtain choroidal thickness data. Six of the studies used the Spectralis SD-OCT (Heidelberg Engineering, Heidelberg, Germany) [14,20,22,23,26,28], three of the studies used the 3D OCT-1000 (Topcon Corporation, Tokyo, Japan) [24,27,29], and one study used the RS-3000 Advance system (NIDEK, Gamagori, Japan) [25]. In addition, two studies used “high-penetration” SD-OCT instruments, which use light sources with a center wavelength of approximately 1050 nm, allowing greater penetration through the RPE and better imaging of the deep choroid compared to the other “standard penetration” SD-OCT instruments mentioned above using light sources centered at the wavelength range of 850–880 nm. One of these two studies used Cirrus HD-OCT (Carl Zeiss Meditec, La Jolla, CA) [15], and the other study used swept-source OCT (Topcon, Inc., Tokyo, Japan) [21]. The characteristics of the studies included are summarized in Table 1.

**Table 1:** Characteristics of the included studies and quality scores on the association between choroidal thickness and CSC status.

Study group / year	Design	Location	Instrument	No. of eyes*	Mean age ^†^ (years)	Refractive errors*	Quality score
Goktas 2014	Prospective	Turkey	Heidelberg Spectralis	20 / 20 / 20	41.2 / 39.3	NA / NA / NA	6
Jirarattanasopa 2012	Prospective	Japan	Topcon SS-OCT	44 / 19 / 17	57.3 / 62.1	-0.8 / NA / 0.1	7
Kang 2013	Retrospective	Korea	Heidelberg Spectralis	16 /—/ 32	48.2 / 45.0	NA / -0.55 / -1.14	7
Kim JH 2013	Retrospective	Korea	Heidelberg Spectralis	40/—/40	46.1 / 65.5	0.13 /—/ NA	7
Kim SW 2011	Retrospective	Korea	Topcon 3D-1000	31 /—/ 29	47.4 / 59.8	-0.6 /—/ 0.18	7
Kim YT 2011	Prospective	Korea	Heidelberg Spectralis	30 / 30 / 30	48.2 / 48.2	-0.5 /—/ -1.3	7
Kuroda 2013	Retrospective	Japan	Zeiss Cirrus HD-OCT	35 /—/ 35	54.4 / 53.9	-0.51 /—/ -0.72	7
Manabe 2015	Prospective	Japan	Nidek RS-3000	22 /—/ 54	55.0 / 54.1	NA /—/ NA	6
Maruko 2011	Retrospective	Japan	Heidelberg Spectralis	66 / 66 / 177	52.8 / 55.6	-1.6 / -1.4 / -1.4	7
Oh 2014	Retrospective	Korea	Topcon 3D-1000	44 / 44 / -	46.5 / NA	-1.4 / -1.3 / -	8
Yang 2013	Prospective	China	Heidelberg Spectralis	15 / 15 / 15	46.0 / 46.5	-0.27 / NA / -0.51	7
Yun 2015	Retrospective	Korea	Topcon 3D-1000	34 / 34 / 34	47.8 / 47.8	-0.79 / -0.81 /-0.49	7

* Central serous chorioretinopathy eyes/unaffected fellow eyes /normal controls

† Central serous chorioretinopathy patients/normal controls;—without unaffected fellow eyes or normal controls

NA, not available.

### Subfoveal Choroidal Thickness

**Fig 2:**
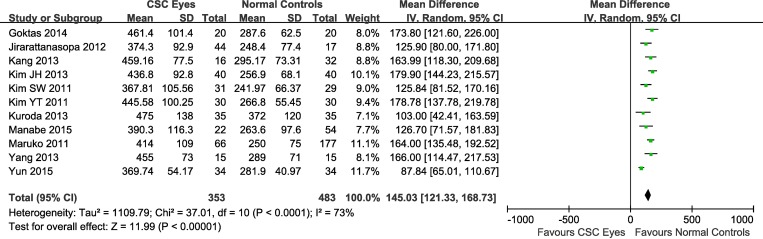
Random-effects model evaluating the association between subfoveal choroidal thickness in CSC eyes and normal control eyes. CSC, central serous chorioretinopathy; SD, standard deviation; IV, inverse variance; CI, confidence interval.

**Fig 3:**
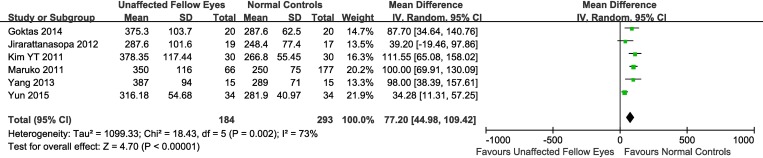
Random-effects model evaluating the association between subfoveal choroidal thickness in unaffected fellow eyes and normal control eyes. SD, standard deviation; IV, inverse variance; CI, confidence interval.

**Fig 4:**
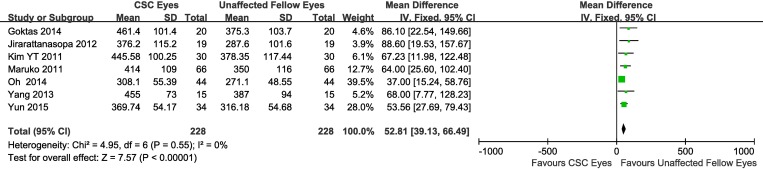
Meta-analysis of the association between subfoveal choroidal thickness in CSC eyes and unaffected fellow eyes. CSC, central serous chorioretinopathy; SD, standard deviation; IV, inverse variance; CI: confidence interval.

When the data from all studies were combined, the mean SFCTs of the affected eyes, unaffected fellow eyes, and normal control eyes were 413.1 ± 93.0 μm, 337.9 ± 90.9 μm, 277.6 ± 73.4 μm, respectively. Adjusting the mean by obtaining a weighted average of SFCTs with weights based on the number of eyes involved in each study resulted in similar values (Table 2). Overall, 11 studies involving 836 eyes compared choroidal thickness of CSC eyes to the thickness in eyes of normal control subjects, 6 studies involving 477 eyes compared choroidal thickness of unaffected fellow eyes of CSC patients to choroidal thickness in eyes of normal controls, and 7 studies involving 456 eyes compared thickness in CSC eyes to thickness in unaffected fellow eyes. As an initial task, we compared the SFCT of the eyes with clinical manifestation of CSC to SFCT of control eyes. This comparison clearly demonstrated that CSC-affected eyes had SFCT which was ~145 μm (34%) bigger compared to normal control’ eyes (weighted mean difference (WMD) = 145.03, 95% confidence interval (CI), 121.33–168.73) and that this difference was highly significant (P<0.00001), with heterogeneity identified. Because of the presence of heterogeneity, a random-effects model was applied to the data (Fig 2). Similarly, the analysis demonstrated that the SFCT of the clinically unaffected fellow eyes was ~77 μm (~29%) bigger compared to the thickness in normal control eyes (WMD = 77.20, 95% CI, 44.98–109.42) and this difference was also highly significant (P<0.00001). Heterogeneity was present in this comparison too, and thus, a random-effects model was applied to the data (Fig 3). Finally, when comparing the mean SFCT of the CSC-affected eyes to the choroidal thickness in unaffected fellow eyes, the analysis demonstrated a subfoveal choroid which was on average ~53 μm (~20%) thicker compared to unaffected fellow eyes (WMD = 52.81, 95% CI, 39.13–66.49) and this difference was also highly significant (P<0.00001), with no heterogeneity identified (Fig 4).

**Table 2:** Mean values and standard deviations of subfoveal choroidal thickness in all studies included in the current meta-analysis.

Study	CSC eyes			Unaffected fellow eyes			Control eyes		
	Mean	SD	n	Mean	SD	n	Mean	SD	n
Goktas 2011	461.4	101.4	20	375.3	103.7	20	287.6	62.5	20
Jirarattanasopa 2012	374.3	92.9	44^*^	287.6	101.6	19	248.4	77.4	17
Kang 2013	459.2	77.5	16	/	/	/	295.2	73.3	32
Kim JH 2013	436.8	92.8	40	/	/	/	256.9	68.1	40
Kim SW 2011	367.8	105.6	31	/	/	/	242.0	66.4	29
Kim YT 2011	445.6	100.3	30	378.4	117.4	30	266.8	55.5	30
Kuroda 2013	475.0	138.0	35^†^	/	/	/	372.0	120.0	35
Manabe 2015	390.3	116.3	22	/	/	/	263.6	97.6	54
Maruko 2011	414.0	109.0	66	350.0	116.0	66	250.0	75.0	177
Oh 2014	308.1	55.4	44	271.1	48.6	44	/	/	/
Yang 2013	455.0	73.0	15	387.0	94.0	15	289.0	71.0	15
Yun 2015	369.7	54.2	34	316.2	54.7	34	281.9	41.0	34
Average	413.1	93.0	/	337.9	90.9	/	277.6	73.4	/
Weighted average^‡^	405.0	96.1	397^§^	332.9	94.9	228^§^	269.4	77.6	483^§^

* - 35% of the eye have bilateral involvement.

† - 29.6% of the eyes have bilateral involvement.

‡—Weighted average was calculated by using weights based on the number of eyes involved in each group.

§—Total number of eyes in that group.

SD, standard deviation; n, number of eyes involved in each group.

### Sub-analysis: Studies Using the Same OCT Equipment

**Fig 5:**
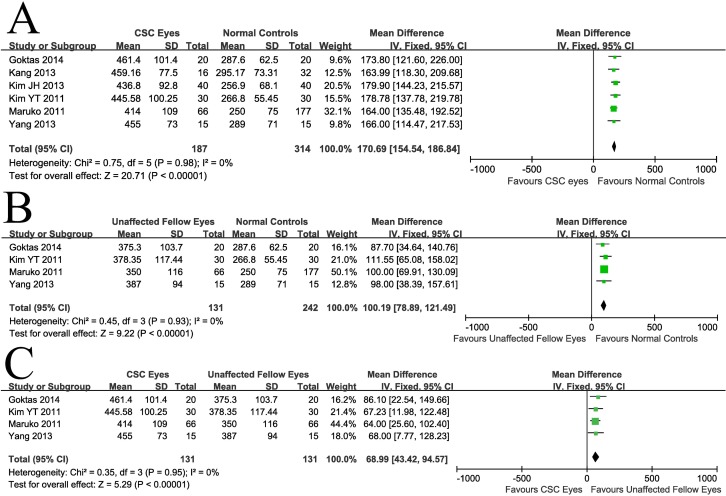
Sub-analysis based on measurement of subfoveal choroidal thickness using Spectralis SD-OCT. **(A)** CSC eyes vs. normal controls, **(B)** Unaffected fellow eyes vs. normal controls, and **(C)** CSC eyes vs. unaffected fellow eyes. CSC, central serous chorioretinopathy; SD, standard deviation; IV, inverse variance; CI, confidence interval.

One limitation of the analysis when all studies are included is the heterogeneity introduced by the use of different OCT instruments, as shown in Table 1. To overcome this problem, we decided to conduct a sub-analysis including only studies that used the same OCT equipment. As the most popular instrument was Spectralis (Heidelberg Engineering), only studies using this instrument were included in the sub-analysis. We hypothesized that restricting the analysis to studies using the same instrument would reduce or even completely eliminate the presence of significant heterogeneity (in the latter case eliminating the need for use of a random-effects model) and would also increase the WMD and reduce the range of the CI of the mean differences. The results of this sub-analysis were very similar to the results of the main analysis: a) the average thickness of subfoveal choroid of the CSC-affected eyes was ~171μm (38%) bigger compared to the thickness of subfoveal choroid in normal control eyes (WMD = 170.69, 95%CI, 154.54–186.84, P<0.00001, Fig 5A); b) the choroid of the CSC-unaffected fellow eyes was ~101 μm (36%) thicker compared to the choroid in normal control eyes (WMD = 100.19, 95%CI, 78.89–121.49, P<0.00001, Fig 5B); c) the choroid of CSC-affected eyes was ~ 69 μm (~16%) thicker compared to the one in unaffected fellow eyes (WMD = 68.99, 95% CI, 43.42–94.57, P<0.00001, Fig 5C). The hypothesized effects of the sub-analysis were confirmed as no heterogeneity was identified in the first two comparisons, the WMD increased in all three comparisons and the CI range decreased in the first (CSC-affected vs. control eyes) and second (CSC-unaffected, fellow eyes vs. control eyes) comparisons. The CI range increased in the third comparison (CSC vs. fellow eyes) probably because of the weight given to one study (Oh et al. 2014) which had a much narrower CI compared to the rest of studies in the main analysis. In addition, Begg’s rank correlation test and Egger’s linear regression test indicated no publication bias for any of the parameters.

## Discussion

In this meta-analysis, we reviewed 12 relevant studies, including a total of 1108 eyes (397 eyes with CSC, 228 unaffected fellow eyes and 483 eyes of normal controls). The results from the group comparisons clearly demonstrated that the subfoveal choroid of the CSC-affected eyes and of the clinically-unaffected, contralateral eyes of the patient groups was thicker compared to the subfoveal choroid of normal control eyes. This was true for all studies included in the main analysis and in the sub-analysis and the difference was very highly significant (P << 0.001).

Despite the considerable progress in increasing our capabilities to investigate retinal structure and function in health and disease especially over the last decade, the true pathophysiology of CSC is still not completely understood. The main location of dysfunction was previously thought to originate in the RPE because one or multiple leakages were seen on fluorescein angiography. However, Negi et al. reported that fluorescein diffused readily into blebs made over damaged RPE, but the subretinal fluid was resorbed more quickly than from blebs overlying normal RPE in rabbits [30]. This result questions whether CSC is caused simply by a passive "leak" from the choroid through the RPE barrier. Currently, the leading hypothesis regarding the pathobiological mechanism of CSC is postulated to be a hyperpermeability of the choroidal vessels and consequent increased choroidal hydrostatic pressure, which leads to accumulation of subretinal fluid [10,11].

Can increased permeability of choroidal blood vessels be associated with a thicker choroid? Iida et al. reported focal choroidal hyperpermeability in 96% of 105 eyes with CSC in Japanese patients in the late phase ICGA [31]. Hyperpermeable choroidal vasculature is thought to produce increased tissue hydrostatic pressure, resulting in pigment epithelial detachments with subsequent RPE defects, which in turn results in focal leakage into the subretinal space [32]. The hyperpermeability might cause choroidal thickening through accumulation of fluid, and the expansion of the choroidal vessels could play a partial role for the choroidal thickening. The current meta-analysis conclusively demonstrates that the subfoveal choroid in CSC eyes is thicker than the choroid in normal control eyes or in unaffected fellow eyes. Maruko et al. reported that although the choroid was thicker in the fellow eyes with choroidal vascular hyperpermeability on ICGA, the choroid was not thicker in the fellow eyes without hyperpermeability [26]. This result indicates a significant correlation between choroidal thickness and choroidal vascular hyperpermeability and may explain better previous findings by Spaide et al. describing choroidal vascular hyperpermeability in fellow eyes of CSC patients based on ICGA [3,33].

It remains unclear whether or not choroidal thickening on OCT may represent solely activity of CSC. One longitudinal follow-up of CSC eyes found that SFCT decreased ~9% after a spontaneous resolution in 16 eyes with spontaneously resolved CSC, but did not return to normal values [22]. Thus, there appears to be a threshold of choroidal thickness above which accumulation of subretinal fluid is much more likely to occur, leading to clinical symptoms of CSC (decreasing in visual acuity, blurring of central vision, double vison, etc.). If this is confirmed based on large, well-designed clinical trials, the assessment of choroidal thickness by SD-OCT may prove to be a useful clinical indictor for early detection, monitoring the progression of evaluating treatment efficacy in CSC. The current meta-analysis also convincingly demonstrated that SFCT was increased not only in CSC eyes, but also in unaffected fellow eyes, suggesting that CSC might be an essentially a bilateral disorder with (mostly) unilateral clinical presentation. The frequency of the bilaterality is reported at 20% to 40% in observational longitudinal studies [4–6]. Broad retinal functional disturbances as assessed by multifocal electroretinography or static perimetry of both eyes in patients with unilateral involvement of CSC also suggest a bilateral functional involvment [34–36]. As the frequency of bilateral cases was high in a long-term follow-up study, it is likely that some unilateral cases of CSC will become bilateral during follow-up. The fact that the mean SFCT in the current meta-analysis was on average ~29% (or ~16% in the sub-analysis) thicker in clinically unaffected fellow eyes compared to normal control eyes, but ~20% (or ~19% in the sub-analysis) thinner compared to CSC-affected eyes (Table 2), supports the notion of a critical threshold in SFCT and a subclinical ‘latent’ phase in CSC in unaffected fellow eyes, that may precede a subsequent clinical manifestation of the disease in both eyes.

**Fig 6:**
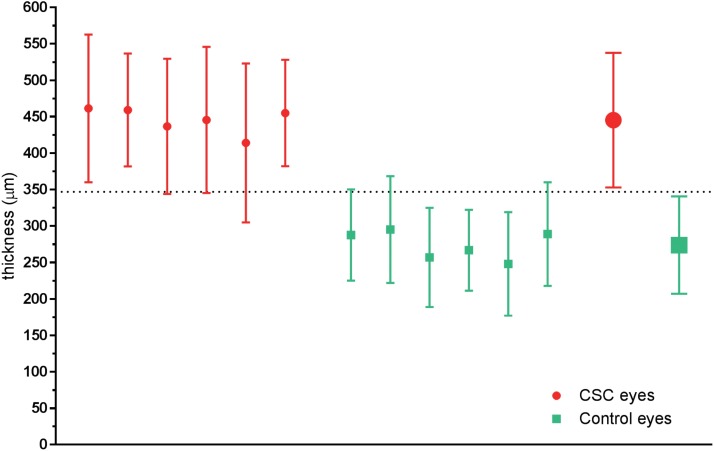
Subfoveal choroidal thickness in control eyes and in eyes with a clinical presentation of CSC determined by using Spectralis SD-OCT. Filled circles represent mean values of subfoveal choroidal thickness (SFCT) in eyes with clinical presentation of CSC, while filled squares represent mean valued of SFCT in control eyes. Error bars indicate standard deviations. Two enlarged symbols in the right end of the figure represent the average SFCT value for each group. The dotted line represents the mid point between the lower end of the standard deviation of CSC-affected eyes and the upper end of the standard deviation of the control eyes. CSC, central serous chorioretinopathy.

Furthermore, the differences in SFCT between the three groups established in the current work may have some clinical significance. It appears that, at least in predominantly East Asian populations, increased SFCT is a risk factor for development of CSC and, therefore, its measurement has the potential to be used as a screening tool. Recently, a threshold value of 395 μm was prosed as an upper limit of normal SFCT based on measurements in French families and literature [37]. The results from our analysis indicate that there is a considerable overlap in absolute values of SFCT between control eyes and eyes with a clinical presentation of CSC on one hand, and clinically-affected CSC eyes and fellow eyes, on the other hand. Because of this overlap, it would be important, especially in the context of a longitudinal follow-up, for the clinician to be aware of a level of SFCT at which the probability of a clinical manifestation of CSC would be considerably increased as it approaches levels characteristic of CSC-affected eyes. One way to define such “threshold of awareness” could be as a mid-point value between the average SFCT of control eyes + 1 SD and the average SFCT of clinically-affected CSC eyes– 1 SD. The least variable result for determining such a threshold could be obtained using the same method of measurement and the same instrument. For example, in our sub-analysis based on studies using only Spectralis SD-OCT, a “threshold of awareness” can be defined as ~347 μm, based on the aggregated results from the six studies selected for the sub-analysis and presented in Fig 5A (dotted line in Fig 6).

SFCT could also be considered as a biomarker in screening for corticosteroid sensitivity, as it has been shown that corticosteroid use can be a predisposing factor for CSC [2,4]. Finally, in several of the studies included in this work, this parameter was used as an indicator of treatment efficacy, and our data support a rational for doing that in addition to other treatment efficacy measures, like visual acuity, multifocal electroretinography, angiography leakage, etc.

This work may have some limitations. First, we cannot fully exclude publication bias. It is possible that some works, especially those published in languages other than English may have been missed. Second, a potential source of heterogeneity is the use of different instruments for choroidal thickness measurement, so the results should be interpreted with caution. However, the results from our sub-analysis based on studies using the same OCT equipment, with no heterogeneity identified, were similar to the results of the main analysis, thus arguing against a significant contribution of the type of instrument used to the differences observed between groups. Third, ethnic backgrounds were almost East-Asians. Although a recent study demonstrated that the difference in SFCT is not statistically significant between Asians, Africans and Caucasians after adjusting for age, refractive error and axial length [38], there has been some controversy regarding the prevalence of CSC in different ethnic groups, with some authors claiming that there is difference [39], while other authors disputing it [40]. Some studies also suggest that there are ethnic differences in the rates of bilateral and multifocal involvement and in severity [4]. Thus, future studies will need to expand to other backgrounds when data become available. Fourth, the mean age of the patients in this analysis was universally in the 40-50s, so our conclusions are valid only for this age group, although some studies have found that age does not have an effect on SFCT when the results are corrected for axial length [41]. Lastly, there are natural interocular variations of choroidal thickness [42], and other factors that can affect choroidal thickness, like smoking, axial length, central corneal thickness, and diastolic ocular perfusion pressure [41,43,44], most of which have not been accounted for in the studies included in the present analysis.

In conclusion, the present analysis firmly establishes an increased SFCT not only in clinically affected eyes with CSC, but also in clinically unaffected fellow eyes in East Asian patients. Despite some limitations, this result provides strong support to the hypothesis that CSC is an essentially a bilateral disorder with mostly unilateral clinical presentation.

## Supporting Information

S1 Checklist PRISMA Checklist. (DOC)
